# Annual Meetings of Hospitals

**Published:** 1920-04-03

**Authors:** 


### Annual Meetings of Hospitals.

### Mount Vernon Hospital.

The Mount Vernon Hospital for Consumption and Diseases of the Chest, henceforth to be known as the Mount Vernon Hospital for Tuberculosis and Diseases of the Lungs and Heart, so it was decided at the annual meeting of Governors held last week, cared for 343 in-patients and 2,330 out-patients during 1919. The accounts show that expenditure last year exceeded income by £4,854. Furthermore, the institution is indebted to its bankers to the extent of £4,000, and has had to sell stock to the value of £2,000 to meet current expenditure. The Committee, however, feels that the hospital now has a brighter future than seemed possible at one time, and believes that it has every reason to anticipate increased support. It does not agree that the substitution of State aid for the voluntary principle would be a gain either to the hospitals or the nation. No State-supported institution could continue its work on the principles upon which the voluntary system was founded. Present accommodation at the hospital is totally inadequate, and the provision of 100 additional beds would meet a very real need.

### The Italian Hospital.

More than half the number of civilian patients cared for at the Italian Hospital last year were British and Colonial, the figures reported at the annual meeting held last week being 282 British and Colonial, and 268 Italian and other nationalities. Of the military patients treated during the same period 200 were British, twelve Canadian, and twelve Australian. The twelve months under review closed with a deficit of £2,730, income being only £5,811 and expenditure £8,541. The Italian Government gave its usual donation of £100. On becoming a patron of the institution last summer, the Prince of Wales wrote to the effect that while serving with the Army in Italy during the war he heard of the splendid work the institution was doing. The average cost of each in-patient per week rose from £2 7s. 6d. in 1918 to £4 3s. 4d. last year, while out-patients cost nearly 3s. 4d. in that period, as compared with 2s. 10d. during the preceding year.

### London Homœopathic Hospital.

Over 2,000 sailors have been treated during the war at the London Homœopathic Hospital, the cost of maintenance, however, exceeding the amount received from the Admiralty by £7,676, which has had to be borne by the institution. Income, it was stated at the annual meeting, held last week, amounted to only £13,017 in 1919, as compared with £22,362 a year previously, while the expenditure of £21,713 in that period had increased to £24,232 during the twelve months under review. The weekly cost of each in-patient has now risen to £3 12s. 7d. The Board feels that the time has now come for doing away with that invidious unfair yet ill-defined distinction of a certain section of the public which is considered to be entitled to treatment on a charitable basis, and for whom are practically reserved the very great advantages of institutional treatment. It points out that in that respect we are a long way behind America, where each patient pays according to his income for exactly similar benefits, and the humblest citizen gets as good medical treatment and nursing as can be obtained by any millionaire. An almoner has been appointed to investigate the circumstances of patients, with a view to securing such contributions to their maintenance as their means permit.

### London Temperance Hospital.

The yearly declension of donations and subscriptions experienced for some long time past has been stayed, says the Board of the London Temperance Hospital in its report, which was adopted at the annual meeting of Governors last week, and a slight increase of support from those sources took place in 1919. Annual subscriptions amounted to £998, as compared with £926 in 1918, while donations advanced from £1,297 to £2,588. The total income of the hospital last year was £11,587, but the expenditure reached £15,000. In-patients to the number of 1,226 were received during the period under review. The number of out-patients treated constituted a record in the history of the hospital, no fewer than 16,466 separate cases having received attention and 82,987 visits paid. Of that number 24,733 have been cured and 12,087 relieved. The number of abstainer in-patients totalled 16,011

